# Pediatric trauma burden in Tanzania: analysis of prospective registry data from thirteen health facilities

**DOI:** 10.1186/s40621-022-00369-7

**Published:** 2022-01-17

**Authors:** Hendry R. Sawe, Sveta Milusheva, Kevin Croke, Saahil Karpe, Juma A. Mfinanga

**Affiliations:** 1 Department of Emergency Medicine, Emergency Medicine Department, MUHAS, Muhimbili University of Health and Allied Sciences, P.O. Box 65001, Dar es Salaam, Tanzania; 2 Department of Emergency Medicine, Muhimbili National Hospital, Dar es Salaam, Tanzania; 3 Development Impact Evaluation Group, World Bank, Washington, DC USA; 4 Harvard T. H. Chan School of Public Health, Boston, MA USA; 5 Lyft, San Francisco, CA USA

**Keywords:** Pediatric injury, Childhood injuries, Tanzania, Africa, Pediatric trauma

## Abstract

**Background:**

Trauma is among the leading causes of morbidity and mortality among pediatric and adolescent populations worldwide, with over ninety percent of childhood injuries occurring in low-income and middle-income countries. Lack of region-specific data on pediatric injuries is among the major challenges limiting the ability of health systems to implement interventions to prevent injuries and improve outcomes. We aim to characterize the burden of pediatric health injuries, initial healthcare interventions and outcomes seen in thirteen diverse healthcare facilities in Tanzania.

**Methods:**

This was a prospective cohort study of children aged up to 18 years presenting to emergency units (EUs) of thirteen multi-level health facilities in Tanzania from 1^st^ October 2019 to 30^th^ September 2020. We describe injury patterns, mechanisms and early interventions performed at the emergency units of these health facilities.

**Results:**

Among 18,553 trauma patients seen in all thirteen-health facilities, 4368 (23.5%) were children, of whom 2894 (66.7%) were male. The overall median age was 8 years (Interquartile range 4–12 years). Fall 1592 (36.5%) and road traffic crash (RTC) 840 (19.2%) were the top mechanisms of injury. Most patients 3748 (85.8%) arrived at EU directly from the injury site, using motorized (two or three) wheeled vehicles 2401 (55%). At EU, 651 (14.9%) were triaged as an emergency category. Multiple superficial injuries (14.4%), fracture of forearm (11.7%) and open wounds (11.1%) were the top EU diagnoses, while 223 (5.2%) had intracranial injuries. Children aged 0–4 years had the highest proportion (16.3%) of burn injuries. Being referred and being triaged as an emergency category were associated with high likelihood of serious injuries with adjusted odds ratio (AOR) 4.18 (95%CI 3.07–5.68) and 2.11 (95%CI 1.75–2.56), respectively. 1095 (25.1%) of patients were admitted to inpatient care, 14 (0.3%) taken to operation theatre, and 25 (0.6%) died in the EU.

**Conclusions:**

In these multilevel health facilities in Tanzania, pediatric injuries accounted for nearly one-quarter of all injuries. Over half of injuries occurred at home. Fall from height was the leading mechanism of injury, followed by RTC. Most patients sustained fractures of extremities. Future studies of pediatric injuries should focus on evaluating various preventive strategies that can be instituted at home to reduce the incidence and associated impact of such injuries.

**Supplementary Information:**

The online version contains supplementary material available at 10.1186/s40621-022-00369-7.

## Background

Injuries are among the major causes of pre-mature and preventable deaths in children across the world (Adeloye et al. 2018). According to the 2020 report of child mortality published by United Nations Inter-agency Group for Child Mortality Estimation, injury is the leading cause of death for children, adolescents and youth aged 5–24 years (United Nations Inter-agency Group for Child Mortality Estimation (UN-IGME). 'Levels Trends in Child Mortality 2020). Children from low and middle-income countries (LMICs) are disproportionately affected, accounting for over 90% of unintentional injury related deaths globally (Tupetz et al. 2020; Peden MM, UNICEF, World 2008; World Health Organization 2021). While there have been efforts in reducing the burden of childhood mortality due to trauma in most high-income countries (HIC), in LMIC, efforts have largely focused on addressing mortality resulting from communicable illnesses, leaving a surge in mortality resulting from injuries among the same age population (Adeloye et al. 2018; Sethi et al. 2017; Ademuyiwa et al. 2012). A recent study on global health estimates of child mortality due to injuries in World Health Organisation (WHO) Europe region found a general decline of injuries over a 15-year period; however, there was persistent inequality between the LMICs and HICs in the region, with a widening gap of injury mortality (Sethi et al. 2017). This trend has been attributed to socio-economic transition in these countries, with more urban dwellers and increasing motor vehicle accidents, combined with a lack of injury prevention strategies (Jullien 2021).

In Sub-Saharan Africa (SSA), injuries are a significant cause of mortality, and for every death, there are thousands of non-fatal injuries, which are likely under-reported, but result in serious disabilities which might be preventable with timely treatment (WHO 2013; Wesson et al. 2017). While the burden of traumatic injury is very high, many of these injuries can be prevented by simple and cost effective interventions that can be instituted at the community level, targeting children and families (Delmira de Sousa Petersburgo CEK 2010; Sleet 2018). The lack of published trauma data (Croke et al. 2020), and specifically pediatric injury data in the region, prevents comprehensive understanding of factors that are associated with pediatric injuries, care processes and outcomes; all of which can support the development of interventions to prevent injury occurrence, post-injury care and long-term rehabilitation care (Wesson et al. 2017; Herbert et al. 2012;  Gallaher et al. 2016).

In Tanzania, like most LMICs, there is paucity of published data on pediatric injury, with few published papers. Most existing literature either focuses on a single cause of injury, such as burn, or they are based on household surveys within a particular geographical context, which limits generalisability of the findings (Simon et al. 2013; Roman et al. 2012; Font et al. 2002; Pérez Méndez et al. 2020). This paucity of data hampers the capacity of health policymakers to understand the burden of pediatric trauma and to prioritize and design effective interventions, such as customizing resources to cater for the needs of management of pediatric injuries (Sleet 2018; Pérez Méndez et al. 2020). In an effort to understand the burden of pediatric injuries, care process and outcome of pediatric injuries in Tanzania, we undertook a study to describe the causes, patterns, interventions and outcomes of pediatric injuries, among patients presenting at the Emergency Units (EUs) of thirteen multi-level health facilities that include the diverse scale of administrative structure of Tanzania’s public health infrastructure.

## Methods

### Study design

**Fig.1:**
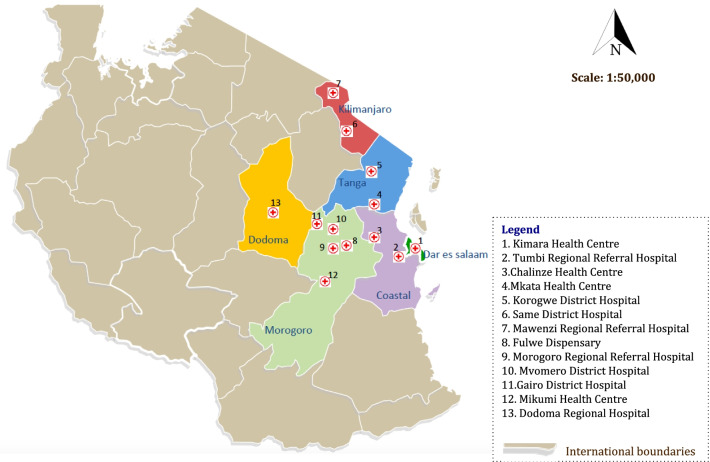
Map of Tanzania showing the location of each health facility

This was a prospective cohort study of children 0–18 years that presented to thirteen multi-level health facility EUs in Tanzania from 1st October 2019 to 30th September 2020. In this study, we implemented trauma registries (TRs) at thirteen health facilities, which include four regional hospitals (*Tumbi, Morogoro, Dodoma and Mawenzi* regional referral hospitals), three district hospitals (*Same, Korogwe and Mvomero* Hospitals), five health centers (*Kimara, Chalinze, Mikumi, Mkata and Gairo* health centers) and one dispensary (*Fulwe* dispensary) (Fig. 1).

### Study setting

This study utilized prospectively collected trauma registry (TR) data from the EU of 13 multi-level health facilities in the United Republic of Tanzania. Tanzania is a lower-middle-income country, with approximately 60 million people, of which over half are aged between 0–19 years (Available from 2021; Bureau and of Statist ics 2016). The public health system operates on a pyramidal model ranging from dispensaries as the lowest level of care to consultant and national level hospitals as the highest level of care (Sirili et al. 2017). There are significant limitations on specialised pediatric neurosurgical trauma care, with such services being available in consultant and national level hospitals. Some regional hospitals have orthopedic and pediatric consultants, but there are substantial limitations around pediatric surgical and orthopedic consultants. All regional and district hospitals have EUs which serve as acute intake areas for patients with acute illness and injuries. While these operate 24 h, seven days a week, there are variable levels of human resource and infrastructure to support care (Sawe et al. 2020). Health centers and dispensaries have dedicated rooms for care of the injured; health centers operate 24 h per day, while dispensaries operate only for 12 h per day. The implementation of TR at these facilities was part of a larger trauma study that was aiming at understanding the health impacts of implementing an Emergency Medical Services (EMS) pilot along the A7 highway that connects Northern to Southern Tanzania (The World Bank 2021). The details and components of pilot implementation of this EMS are discussed elsewhere (Sawe et al. 2021). The TR was set up to enroll all injured patients at EUs of 13 public health facilities that are within two kilometers of the highway, which involved 6 health facilities (which were part of the pilot EMS implementation): 2 regional hospitals (*Tumbi and Morogoro*), 3 health centers (*Kimara, Chalinze and Mikumi*) and 1 dispensary (*Fulwe)*. The 7 additional (comparison group) facilities included 2 regional hospitals (*Dodoma and Mawenzi*), 3 district hospitals (*Same, Korogwe and Mvomero*) and 2 health centers (*Mkata and Gairo*), all of which were located on a different though comparable highway. As part of the main study one of the treatment facilities was a dispensary, which functioned more like a health center given the nature and volume of patients presenting to this facility, and therefore, it was better matched with a health center in a comparison group.

### Study population

Children aged 0 to 18-years-old presenting to any of thirteen health facilities with trauma related complaints, either from the scene of injury or as referral from lower facilities, were eligible for inclusion in the study. We excluded children returning to the EU for follow up care after initial interventions or those who were referred from one of the sites involved in the study.

### Data source

A paper-based standardized trauma documentation form was implemented at the EU of each of thirteen health facilities and was used for both clinical care as well as informing the TR. This standardized trauma form was initially adopted and modified from the World Health Organisation (WHO) standardized trauma form (WHO 2020). Prior to implementation, the paper-based trauma documentation form was modified and re-piloted to ensure inclusion of additional variables of interest, especially related to road traffic injuries (RTI). The final version of the form-included variables related to demographics, injury location, clinical presentation, injury details, injury severity, level and number of injuries, management, care outcomes, diagnosis, consultation and final disposition of the patients. The standardized trauma form was printed with a carbonless copy to allow the duplication of information for clinical documentation as well as retaining a copy that was used for abstracting data to inform the TR. Data from the standardized trauma form were abstracted to an online data capture software Research Electronic Data Capture (REDCap) (© REDCap version 7.2.2, Vanderbilt, Nashville, TN, USA).

### Study procedure

We recruited and trained a research assistant (RA) and trauma data coordinator (TDC) in each health facility to support data collection. In order to ensure optimal coverage, regional level hospitals had two RAs, while the rest of health facilities had one RA. The TDCs were health care providers in these facilities and had an overall clinical oversight responsibility to ensure compliance and quality of clinical documentation by the clinicians, as well as regular data collection procedures. Prior to launching data collection, we trained RAs and TDCs on the overview of primary trauma care and documentation of variables in the standardized trauma form as well as abstracting data to the REDCap tool using digital tablets.

All patients presenting to the EU of these health facilities with injury related complaints were manually recorded into the trauma forms by clinicians and RAs. Details of the care process for the patients were expected to be documented by the clinician, while the RA supported the documentation as well as abstracting the information to the REDCap at the end of the patient care process. Clinicians filled out the form 24 h a day as part of the documentation process when seeing any patient presenting for trauma. This allowed for continuous data collection, and RAs would enter the forms during their working hours. The incoming data were regularly monitored for logical inconsistencies, missing data, outliers, as well as the capture rate per health site. Furthermore, the study authors received copies of completed trauma forms from each site, and performed checks on a randomly selected sample of forms, independently entering the data from the paper form and comparing all variables to those entered by the RAs and provided regular feedback to the TDCs and RAs.

### Data analysis

Data were exported from REDCap and imported onto the Statistical Package for the Social Sciences (SPSS version 22.0, IBM, Ltd, Carolina, USA)*,* cleaned, coded and analyzed. The descriptive statistics of trauma patients were summarized by frequency distribution tables of patient demographics, and mean and standard deviation or median and interquartile range (IQR). Variables analyzed included mechanism of injury, injury intent, triage level, referral pattern and final EU disposition. The final EU diagnoses were coded using International Classification of Diseases (ICD)-10. Logistic regression was performed in order to understand the risk factors for potentially serious injuries (those that necessitated ward, ICU, operating theatre admission or referral to other facilities).

## Results

### Patient characteristics

Among 18,553 trauma patients seen in all thirteen-health facilities during the study period, 4368 (23.5%) were children, of whom 2894 (66.7%) were male, and overall median age was 8 years (Interquartile range 4–12 years). Motorized (two or three) wheeled vehicles 2401 (55%) were the most common mode of EU arrival; 3748 (85.8%) arrived at EU directly from the injury site. Overall 3223 (73.8%) patients were triaged as priority cases. Table 1

**Table 1:** Patient characteristics

	*N* = 4368
Sex*	
Male	2894 (66.3%)
Female	1447 (33.1%)
Age^ň^	
Median (IQR) years	8 years (IQR: 4–12 years)
Age groups	n (%)
< 5 years	1165 (26.7)
5–9 years	1433 (32.9)
10–14 year	1040 (23.9)
15- < 18 years	676 (15.5)
Education level^γ^	
No formal education	1100 (25.2)
Primary school	2432 (55.7)
Secondary school	553 (12.7)
Vocational education	13 (0.3)
College/ University	7 (0.2)
Triage level^*Ħ*^	
Emergency	651 (14.9)
Priority	3223 (73.8)
Queue	430 (9.8)
Referral status	
Direct from injury site	3748 (85.8)
Referred	519 (11.9)
Unknown	101 (2.3)
Mode of arrival	
Motorcycle	1852 (42.4)
Tricycle	549 (12.6)
Ambulance	94 (2.2)
Bicycle	20 (0.5)
Walk-in	681 (15.6)
Car	524 (12.0)
Bus	10 (0.2)
Mini-bus	502 (11.5)
Other	62 (1.5)
Unknown	74 (1.7)

^*^27 (0.6%) had undocumented gender status

^ň^54 (1.2%) had missing age

^Ħ^ 64 (1.5%) had missing triage category

^γ^263 (6.9%) had missing education level

### Mechanism, injury intent and place of injury

Fall from height was the most commonly reported mechanism of injury in the study population, occurring in 1592 (36.4%) of children. Road traffic injuries were the most common mechanism (31.2%) in children aged 15 to 18 years of age. Overall most injuries occurred at home (56.0%), and the majority of injuries (82.1%) were unintentional. Table 2

**Table 2:** Mechanism, intent and injury settings

Variables*	Age distribution (in years)				
Mechanism**	Overall (*N* = 4368)	0–4 (*n* = 1165)	5–9 (*n* = 1433)	10–14 (*n* = 1040)	15- < 18 (*n* = 676)
	*n* (%)	%	%	%	%
Fall from height	1592 (36.4)	41.4	42.9	32.6	20.3
RTC	840 (19.2)	12.4	17.5	22.1	31.2
Stab or cut	476 (10.9)	5.5	11.7	14.1	13.3
Animal bite	343 (7.9)	4.9	9.5	9.9	6.8
Burn	291 (6.7)	16.2	4.0	1.4	2.5
Blunt trauma	200 (4.6)	3.1	3.7	5.8	7.1
Hit by falling object	120 (2.7)	3.0	2.7	2.7	2.8
Sexual assault	80 (1.8)	0.4	1.4	2.4	4.4
Poisoning	38 (0.9)	1.7	0.1	0.5	1.5
Others^*Ħ*^	230 (5.3)	6.8	7.5	4.3	7.0
Injury intent					
Intentional	147 (3.4)	0.8	1.8	4.8	8.7
Unintentional	3586 (82.1)	85.2	83.3	82.2	74.0
Unknown	635 (14.5)	14.1	14.9	13.0	17.3
Location of injury					
Urban	2468 (56.5)	659 (56.6)	835 (58.3)	562 (54.0)	397 (58.7)
Rural	1633 (37.4)	440 (37.8)	507 (35.4)	413 (39.7)	239 (35.4)
Unknown	267 (6.1)	66 (5.7)	91 (6.4)	65 (6.3)	40 (5.9)
Place of injury					
Home	2445 (56.0)	77.2	58.7	44.0	30.3
On the Road	975 (22.3)	12.8	21.2	26.5	35.7
School	355 (8.1)	1.6	8.9	12.6	11.4
Work	159 (3.6)	3.9	2.9	3.9	4.3
Playground	107 (2.5)	0.1	1.6	4.0	6.1
Public Space	252 (5.8)	3.5	5.1	6.9	9.9
Others	11 (0.3)	0.2	0.1	0.4	0.6
Unknown	64 (1.5)	0.9	1.4	1.6	1.8

^*^ 54 (1.2%) had missing age

^**^158 (3.6%) had unknown or missing documentation of mechanism of injury

^Ħ^Others includes gunshot wound, foreign body inhalation, suffocation, drowning

When looking at mechanisms of injury by health facilities, we found that regional hospitals had the highest proportion of patients with fall (43.9%) and RTC (22.1%) related injuries, while Health Centers had the highest proportion of stab or cut injuries (17.4%), animal bite (9.4%) and burn related injuries (8.1%) of which majority 275 (94.5%) occurred at home, involving touching hot liquid or food. Table 3.

**Table 3:** Mechanism of injury by health facility

Mechanism of injury**	All	Regional hospitals	District hospitals	Health centers*
	*N* = 4368	*N* = 2171	*N* = 502	*N* = 1695
	*n* (%)	%	%	%
Fall	1592 (36.4)	43.9	33.5	27.8
Road Traffic Crash	840 (19.2)	22.1	20.3	15.3
Stab or cut	476 (10.9)	6.4	8.4	17.4
Animal Bite	343 (7.9)	6.8	7.4	9.4
Burn	291 (6.7)	5.9	5.0	8.1
Blunt force trauma	200 (4.6)	3.6	2.6	6.4
Hit by falling object	120 (2.7)	2.4	4.0	2.8
Sexual Assault	80 (1.8)	1.8	3.6	1.4
Poisoning	38 (0.9)	0.8	1.0	0.9
Drowning	23 (0.5)	0.3	2.0	0.4
Suffocation	11 (0.3)	0.4	0.2	0.1
Gunshot	2 (0.05)	0.0	0.2	0.0
Other	194 (4.4)	4.6	5.2	4.1

^*^Includes one dispensary

^**^158 (3.6%) had unknown or missing documentation of mechanism of injury

### ICD-10 diagnosis by age group and mechanism of injury

Multiple superficial injuries (14.4%), fracture of forearm (11.7%) and open wounds (11.1%) were the top three diagnoses and accounted for over one-third of diagnoses. Intracranial injuries accounted for 5.2% of the overall diagnoses. Dog bites (5.3%) were more common in the age group 5–9 years, while maltreatment syndrome (4.6%) was more common in the age group 15 to 18 years. Table 4. Falls resulted in most extremity fractures, accounting for three quarters of the fractures of forearm (84.8%), as well as fracture of shoulder and upper arms (84.9%), while RTCs were responsible for over half (61.4%) of intracranial injuries Additional file 1.

**Table 4:** ICD-10 diagnosis by age group

ICD-10 diagnosis**	Age distribution (in years)				
	Overall* (*N* = 4314)	0–4 (*n* = 1165)	5–9 (*n* = 1433)	10–14 (*n* = 1040)	15- < 18 (*n* = 676)
	*n* (%)	%	%	%	%
Multiple superficial injuries, unspecified	622 (14.4)	13.1	12.1	16.2	18.5
Fracture of forearm	505 (11.7)	7.9	14.0	13.7	9.9
Open wound of unspecified body region	481 (11.1)	6.4	12.2	13.3	12.3
Fracture of shoulder and upper arm	385 (8.9)	9.4	12.8	6.3	3.7
Burns and corrosions	296 (6.9)	16.3	3.9	1.5	3.0
Open wounds involving multiple body regions	232 (5.4)	4.4	5.0	5.9	7.0
Bitten or struck by dog	228 (5.3)	3.4	7.4	5.3	3.8
Fracture of lower leg, including ankle	226 (5.2)	3.9	5.7	5.7	5.9
Intracranial injury	223 (5.2)	5.7	4.8	4.3	5.9
Fracture of femur	207 (4.8)	5.8	4.8	4.0	3.3
Maltreatment syndrome	79 (1.8)	0.5	1.2	2.4	4.6
Effects of foreign body entering through natural orifice	70 (1.6)	4.0	1.1	0.7	0.0
Bitten or struck by other mammals	69 (1.6)	0.3	0.9	3.6	2.2
Sprain and strain of other and unspecified parts of foot	68 (1.6)	2.5	1.3	1.3	1.0
Fracture at wrist and hand level	67 (1.6)	1.1	1.2	2.0	2.4
Fracture unspecified	56 (1.3)	1.4	1.0	1.9	0.7
Dislocation, sprain and strain of joints and ligaments of elbow	38 (0.9)	1.1	1.3	0.6	0.1
Contact with hornets, wasps and bees	34 (0.8)	1.1	0.7	0.9	0.3
Dislocation unspecified	34 (0.8)	1.1	0.8	0.7	0.1
Poisoning by, adverse effects of and under dosing of drugs	33 (0.8)	1.4	0.1	0.3	1.8

^*^Overall 54 patients were missing age. **These are top 20 EU diagnoses, only primary diagnosis was included, and 153 patients were missing final EU diagnosis

### Nature of injuries with risk factors for serious injuries

Overall, male patients with AOR 1.17 (95%CI 1.01–1.35) and those in age group 0 to < 5 years (AOR: 1.44 (95%CI 1.14–1.81) had significantly higher likelihood of having serious injuries requiring hospital admission or referral. Being referred and having an initial triage level as an emergency was associated with high likelihood of serious injuries with AOR 3.17 (95%CI 2.57–3.90) and 4.18 (95%CI 3.07–5.68), respectively. Table 5

**Table 5:** Risk factors for serious injuries

Variable	Overall	Non-discharged*	Unadjusted OR	Adjusted OR	*p *value
	*N* = 4368	*n* (%)	UOR [95% CI]	AOR [95% CI]	
Mechanism of injury					
Fall	1592	678 (42.6)	1.92 (95%CI 1.42–2.61)	1.93 (95%CI 1.42–2.63)	< 0.0001
RTC	840	386 (45.9)	2.20 (95%CI 1.60–3.03)	2.35 (95%CI 1.69–3.28)	< 0.0001
Stab or cut	476	31 (6.5)	0.18 (95%CI 0.11–0.29)	0.24 (95%CI 0.15–0.38)	< 0.0001
Animal bite	343	36 (10.5)	0.30 (95%CI 0.19–0.48)	0.38 (95%CI 0.24–0.61)	< 0.0001
Burn	291	150 (51.5)	2.76 (95%CI 1.91–3.99)	3.00 (95%CI 2.01–4.49)	< 0.0001
Blunt force trauma	200	32 (16.0)	0.49 (95%CI 0.31–0.79)	0.57 (95%CI 0.35–0.93)	0.025
Hit by falling object	120	26 (21.7)	0.72 (95%CI 0.43–1.21)	0.85 (95%CI 0.49–1.46)	0.559
Sexual assault	80	23 (28.8)	1.05 (95%CI 0.59–1.84)	1.30 (95%CI 0.73–2.35)	0.368
Poisoning	38	18 (47.4)	2.33 (95%CI 1.16–4.69)	2.87 (95%CI 1.39–5.89)	0.004
Others	230	64 (27.8)	Ref	Ref	
Age^ň^	*N* = 4368				
< 5 years	1165	446 (38.3)	1.45 (95%CI 1.18–1.77)	1.44 (95%CI 1.14–1.81)	0.002
5–9 years	1433	470 (32.8)	1.13 (95%CI 0.93–1.39)	1.12 (95%CI 0.9–1.41)	0.3
10–14 year	1040	330 (31.7)	1.08 (95%CI 0.88–1.34)	0.98 (95%CI 0.77–1.24)	0.849
15–18 years	676	203 (30.0)	Ref	Ref	
Referral^*Ħ*^	N = 4267				
Direct from injury site	3748	1142 (30.5)	Ref	Ref	
Referred	519	308 (59.3)	3.33 (95% CI 2.76–4.02)	3.17 (95%CI 2.57–3.90)	< 0.0001
Triage level ^ň^	*N* = 4304				
Emergency	651	334 (51.3)	3.93 (95%CI 2.97–5.18)	4.18 (95%CI 3.07–5.68)	< 0.0001
Priority	3223	1033 (32.1)	1.76 (95%CI 1.38–2.24)	1.89 (95%CI 1.45–2.49)	< 0.0001
Queue	430	91 (21.2)	Ref	Ref	
Injury intent^*ρ*^	*N* = 3733				
Intentional injuries	147	50 (34.0)	Ref	Ref	
Unintentional injuries	3586	1171 (32.7)	0.98 (95%CI 0.64–1.39)	0.99 (95%CI 0.67–1.48)	0.99
Gender**	*N* = 4341				
Male	2894	1008 (34.8)	1.16 95%CI (1.01–1.33)	1.17 (95%CI 1.01–1.35)	0.041
Female	1447	456 (31.5)	Ref	Ref	

^*^Included injuries requiring hospital admission, operation theatre procedure or referral to higher level of care

^ň^54 (1.2%) had missing age, **27 (0.6%) had undocumented gender status, ^Ħ^ 101 had unknown referral status

^ň^Emergency: to be seen immediately, and, priority: to be seen within a few minutes of arrival

^ρ^In 635, (14.5%) patients intention of injury was unknown

### Final EU disposition by age group

Most patients 2829 (64.8%) were discharged from the EU, while 28.2% of children aged 0–4 years were admitted to inpatient care, and 10.0% were referred to higher-level hospitals. The overall EU mortality was 25 (0.6%), and 1.0% of patients aged 5 to 9 years died while receiving care in the EU. Table 6

**Table 6:** Final EU disposition by age group

Disposition*	Age distribution (in years)				
	Total *N* = 4368 ^ň^	0–4 *n* = 1165	5 to 9 *n* = 1433	10 to 14 *n* = 1040	15 to 18 *n* = 676
	*n* (%)	%	%	%	%
Discharged home	2829 (64.8)	60.3	65.5	66.8	68.5
Admitted to ward	1095 (25.1)	28.2	23.4	23.8	24.6
Transferred to another facility	363 (8.3)	10.0	8.9	7.5	5.2
Died at ED	25 (0.6)	0.4	1.0	0.3	0.4
Admitted to Operating Theatre (OT)	14 (0.3)	0.1	0.5	0.4	0.3
Unknown	42 (1.0)	1.0	0.8	1.2	1.0

^ň^54 (1.2%) had missing age

## Discussion

This study used prospectively collected injury surveillance data from a novel trauma registry implemented at thirteen multilevel health facilities over a period of one year to provide a detailed picture of pediatric injuries treated at health facilities in Tanzania. The findings from this study will provide an evidence base for implementation of appropriate injury prevention strategies, as well as guiding resource allocation to inform care processes at these facilities and other similar settings. In Tanzania, most prior studies on pediatric trauma have been limited to single tertiary sites and have spanned less than one year (Sirili et al. 2017; Sawe et al. 2020). The multisite nature of implementation of this trauma registry involving multilevel facilities with diverse resources, level of care and geographical location, provides a broader picture of the description of burden of pediatric injuries and its associated outcomes in Tanzania.

In these health facilities, pediatric injuries accounted for nearly one-quarter of all trauma cases, highlighting the public health importance of this problem. The peak incidence for injuries in this study is 5 to 9 years of age which is in agreement with prior studies done in similar settings (Sirili et al. 2017; The World Bank 2021). Inability of children to recognize and avoid potential injury risks on their own is one of the unique risks that young children face (Sawe et al. 2021). While the observed high incidence of injury in this age group underscores this fact, it further provides an opportunity for targeted interventions in specific injury settings to reduce the risk and severity of injury (Delmira de Sousa Petersburgo CEK 2010). Similar to previous studies, male children were more affected than females, with a male to female ratio of 2:1. We did not evaluate further the reasons behind this difference, however prior studies have attributed this male preponderance to the overactive nature of male children as compared to female ones (WHO 2020).

In this cohort, one-third of patients were either admitted or referred to higher-level health facilities, with children in the age group 0 to 4 years having the highest proportion of those admitted or referred, compared to other age groups. We evaluated the risk factors for serious injuries, defined by the need for hospitalization, emergency operation or transfer to higher level facilities. Patients who arrived to the EU as referral cases from lower facilities had a significantly higher likelihood of serious injuries compared to those who arrived directly from the injury site. This is likely due to the fact that these patients who are referred to the EU have sustained serious injuries that could not be managed by the lower-level facilities due to resources or expertise limitations, and hence necessitated the transfer to higher facilities. Similarly, when a patient is triaged to an emergency category, they had a two-fold likelihood of serious injuries compared to other triage categories. Poisoning as a mechanism of injury was found to be associated with a 2.9-fold likelihood of serious injuries. There is a lack of dedicated poison treatment centers in Tanzania (Considine et al. 2001), which limits the ability of most facilities to appropriately manage patients with suspected poisoning. Characterizing the nature and severity of poisoning will be key to understanding the resources and expertise needed to inform development of this center in Tanzania and other LICs.

In Tanzania, there is a lack of formal pre-hospital service, which limits the ability of the health system to provide holistic emergency medical services (EMS) to acutely ill and injured patients (Simon et al. 2013). In this study, over three-quarter of patients presented to the EU directly from the injury sites, and the majority used motorized (two or three) wheeler as mode of transport to the EU. The absence of formalized pre-hospital care is not only limiting effective EMS provision, but predisposes injured patients to delay of care access, as well as potential for secondary injuries due to improper handling of victims of injuries (Museru et al. 2004).

In the EU, the majority of patients were triaged to either priority or emergency levels of care, requiring immediate life saving interventions to save their lives or prevent lifelong disabilities. In patients presenting with acute illness or injuries, the initial triage level has been associated with both level of resource utilization as well as the clinical outcomes of patients (Gome et al. 2005). These findings indicate the need to further evaluate the outcome, resources availability and referral patterns of pediatric patients based on the initial level of triage which can be used in guiding the development of protocols for care and referral.

Most injuries were unintentional, occurred at home and resulted from a fall as the main mechanism of injury, which is in concordance with prior studies (Adesunkanmi et al. 1998). Playing has been described as the most common activity at the time of domestic injuries in pediatric patients (Stracciolini et al. 2014). Home-based safety interventions have been shown to reduce injury related admissions in HICs (Hill et al. 2021; Watson and Errington 2016). Given that most injuries in this study occurred at home, a setting that is usually perceived to be safe by parents, plans to reduce the incidence of home related injuries would require devising and testing context appropriate home-safety interventions at different stages of children's growth. Interestingly, when we analyzed the age specific mechanisms of injury, we found that as the age increases (from 0–4 years to 15—< 18 years) the settings change from home to the road, as well as increase in the proportion of intentional injuries. We believe that this is due to the fact that with increasing age, there is a likelihood of children taking part in daily activities and outdoor sports independently and hence predisposing themselves to risk of RTC and other outdoor injuries, as well as intentional injuries (Mbarouk et al. 2017; Kuzma et al. 2015).

Fracture of the forearm, fracture of shoulder and fracture of the upper arm accounted for the majority fractures of extremities, a finding that is in concordance with most of the previously published studies of pediatric injuries (Kuzma et al. 2015). The peak incidence of injuries of extremities was at the age group of 5 to 10 years, which corresponds to our observed high incidence of fall as a mechanism of injury recorded in this age group. In previously published studies, intracranial injuries have been associated with poor clinical progression and outcomes (Kuzma et al. 2015). In our study population, intracranial injuries accounted for five percent of the final EU diagnosis with an equal distribution across all age groups. Evaluation of the long-term sequelae of these injuries will enable understanding of resources and capacity at each of these facilities, as well as the associated outcomes. Similar to previously published studies (Ahn et al. 2011 Aug), burn injuries were found to be fairly common in this study population and were mostly concentrated in the age group 0–4 years, which had 4–11 times higher incidence compared to other age groups. In this age group, nearly all burn injuries occurred at home, with over ninety percent resulting from touching hot liquid or food. Community level education on prevention of accidental burns at home targeting heads of household, with focus on cost-effective infrastructure improvement, and child caregivers can have impact on reducing the incidence burn injuries in this age group (Outwater et al. 2013).

## Limitations

This study included patients from a purposefully selected sample of health facilities that were located close to strategically busy highways in Tanzania, and hence may limit its generalisability to the rest of health facilities in the country. However, we believe the multilevel nature of these health facilities, ranging from lower (dispensary) to the tertiary (regional hospital) level has provided an opportunity to better understand the burden of pediatric injuries at these different levels. The overall TR capture rate was not 100%, we had specific gaps in capturing patients who were dead on arrival at the facility. These patients did not pass through the emergency medical department at most facilities but were taken directly to the mortuaries, which resulted in the low capture rate for some facilities. Furthermore, the COVID-19 pandemic impacted our overall data collection process, as we had to withdraw research assistants and have them work from home to minimize risk of disease transmission, hence affecting the quality of data collection. However, using a standardized clinical documentation form filled out by clinicians ensured that care to the patient and data collection was maintained during the study period.

## Conclusions

In these multilevel health facilities in Tanzania, pediatric injuries accounted for nearly one-quarter of all injuries. Over half of injuries occurred at home, fall from height was the leading mechanism of injury, followed by RTC. Only two percent of patients arrived at EU by ambulance. Patients who arrived to the EU as referral cases, those triaged as emergency category had more than two-fold likelihood of serious injuries requiring hospitalization, emergency operation or transfer to higher levels of care. Future studies of pediatric injuries should focus on evaluating various preventive strategies that can be instituted at home to reduce the incidence and associated impact of such injuries, as well as evaluation of transport systems between healthcare facilities or from the scene and consideration for development of a formal emergency medical service system.

## Supplementary Information

**Additional file 1: Supplementary Table 1**. Mechanism of injury by health facility.** Supplementary Table 2**. Gander by mechanism of injury.

## Data Availability

The datasets used and/or analyzed during the current study are available on request from Principal Administrators.

## Declarations

### Permission to publish

The permission to publish was obtained from the National Health Research Ethics Review Committee (NatREC).
