# Supplementary material for: Pediatric trauma burden in Tanzania: analysis of prospective registry data from thirteen health facilities
Source: Inj Epidemiol. 2022 Jan 17;9:3. doi: 10.1186/s40621-022-00369-7 (PMC8762441; doi:10.1186/s40621-022-00369-7)
Supplement: Supplementary file 1 — Additional file 1: Supplementary Table 1. Mechanism of injury by health facility. Supplementary Table 2. Gander by mechanism of injury. [file 40621_2022_369_MOESM1_ESM.docx]

**Supplementary Table 1: Mechanism of injury by health facility**

|  | Overall | Chalinze | Dodoma | Fulwe | Gairo | Kimara | Korogwe | Mawenzi | Mikumi | Mkata | Morogoro | Mvomero | Same | Tumbi |
| --- | --- | --- | --- | --- | --- | --- | --- | --- | --- | --- | --- | --- | --- | --- |
|  | N=4368 | N=333 | N=467 | N=238 | N=258 | N=490 | N=111 | N=423 | N=121 | N=255 | N=765 | N=171 | N=220 | N=516 |
| **Mechanism of injury** | n (%) | % | % | % | % | % | % | % | % | % | % | % | % | % |
| Fall | 1592 (36.4) | 21.0 | 47.3 | 18.9 | 33.3 | 36.1 | 34.2 | 32.6 | 31.4 | 21.6 | 46.0 | 29.8 | 35.9 | 46.9 |
| Road Traffic Crash | 840 (19.2) | 14.7 | 22.5 | 12.2 | 18.2 | 14.3 | 24.3 | 18.0 | 19.0 | 16.1 | 23.4 | 15.2 | 22.3 | 23.1 |
| Stab or cut | 476 (10.9) | 13.2 | 4.7 | 27.7 | 12.0 | 18.0 | 3.6 | 9.0 | 16.5 | 18.0 | 6.3 | 11.1 | 8.6 | 6.0 |
| Animal Bite | 343 (7.9) | 8.7 | 12.6 | 5.9 | 11.6 | 7.8 | 5.4 | 9.2 | 8.3 | 14.9 | 3.4 | 12.3 | 4.5 | 4.5 |
| Burn | 291 (6.7) | 11.4 | 1.9 | 8.4 | 10.5 | 6.5 | 5.4 | 7.3 | 13.2 | 2.0 | 9.3 | 6.4 | 3.6 | 3.3 |
| Blunt force trauma | 200 (4.6) | 2.7 | 1.9 | 13.0 | 8.9 | 7.3 | 1.8 | 6.9 | 2.5 | 2.7 | 2.5 | 3.5 | 2.3 | 4.1 |
| Hit by Falling Object | 120 (2.7) | 7.5 | 2.8 | 1.7 | 2.7 | 1.6 | 1.8 | 2.8 | 1.7 | 0.4 | 2.4 | 5.8 | 3.6 | 1.9 |
| Sexual Assault | 80 (1.8) | 2.4 | 2.8 | 1.3 | 0.4 | 1.6 | 0.9 | 2.8 | 0.0 | 1.6 | 0.9 | 7.6 | 1.8 | 1.2 |
| Suffocation | 11 (0.3) | 0.3 | 0.0 | 0.0 | 0.0 | 0.0 | 0.0 | 0.9 | 0.8 | 0.0 | 0.5 | 0.0 | 0.5 | 0.0 |
| Poisoning | 38 (0.9) | 0.6 | 0.9 | 0.8 | 1.9 | 0.4 | 0.0 | 0.7 | 1.7 | 1.2 | 0.7 | 1.8 | 0.9 | 1.0 |
| Gunshot | 2 (0.05) | 0.0 | 0.0 | 0.0 | 0.0 | 0.0 | 0.0 | 0.0 | 0.0 | 0.0 | 0.1 | 0.0 | 0.5 | 0.0 |
| Drowning | 23 (0.5) | 0.6 | 0.4 | 0.4 | 0.0 | 0.2 | 9.0 | 0.2 | 1.7 | 0.0 | 0.1 | 0.0 | 0.0 | 0.6 |
| Others | 194 (4.4) | 10.2 | 1.1 | 2.5 | 0.0 | 4.3 | 2.7 | 7.6 | 0.8 | 2.7 | 4.2 | 4.1 | 7.3 | 5.8 |

***158 (3.6%) had unknown or missing documentation of mechanism of injury*

**Supplementary Table 2: Gander by mechanism of injury**

|  | Overall | Female | Male |
| --- | --- | --- | --- |
|  | N=4368 | N=1447 | N=2894 |
| **Mechanism of injury** | % | % | % |
| Fall | 1592 (36.4) | 31.6 | 39.1 |
| Road Traffic Crash | 840 (19.2) | 18.2 | 19.8 |
| Stab or cut | 476 (10.9) | 10 | 11.3 |
| Animal Bite | 343 (7.9) | 9.3 | 7.1 |
| Burn | 291 (6.7) | 8.5 | 5.7 |
| Blunt force trauma | 200 (4.6) | 3.7 | 5.0 |
| Hit by Falling Object | 120 (2.7) | 2.8 | 2.7 |
| Sexual Assault | 80 (1.8) | 3.9 | 0.8 |
| Suffocation | 11 (0.3) | 1.5 | 0.6 |
| Poisoning | 38 (0.9) | 0.5 | 0.1 |
| Gunshot | 2 (0.05) | 0 | 0.1 |
| Drowning | 23 (0.5) | 0.4 | 0.6 |
| Other | 194 (4.4) | 5.7 | 3.8 |

**Supplementary Table 3: ICD-10 diagnosis by Mechanism of Injury**

|  | **Mechanism of Injury** | | | | | | | | | |
| --- | --- | --- | --- | --- | --- | --- | --- | --- | --- | --- |
|  | **Overall** | **Fall** | **RTC** | **Stab/Cut** | **Animal Bite** | **Burn** | **Blunt force** | **Hit by Falling Object** | **Sexual Assault** | **Others*** |
| **ICD-10 diagnosis** | N | % | % | % | % | % | % | % | % | % |
| Superficial injuries involving multiple body regions | 622 | 25.7 | 36.7 | 10.9 | 1.6 | 0.3 | 12.9 | 3.9 | 0 | 5.0 |
| Fracture of forearm | 505 | 84.8 | 7.9 | 0.2 | 0.2 | 0.2 | 1.2 | 0.8 | 0.2 | 3.4 |
| Open wound of unspecified body region | 481 | 11.2 | 12.3 | 51.8 | 1.5 | 0.4 | 4.2 | 6.4 | 0 | 9.4 |
| Fracture of shoulder and upper arm | 385 | 84.9 | 9.4 | 0.8 | 0 | 0.0 | 1.0 | 0.3 | 0 | 2.9 |
| Burns and corrosions | 296 | 0.7 | 0.7 | 0 | 0 | 87.5 | 0 | 0.3 | 0 | 8.4 |
| Open wounds involving multiple body regions | 232 | 20.7 | 21.1 | 28.9 | 3.0 | 2.2 | 5.2 | 7.8 | 0.9 | 5.2 |
| Bitten or struck by dog | 228 | 0 | 0 | 0 | 95.1 | 0 | 0 | 0 | 0 | 2.2 |
| Intracranial injury | 223 | 21.1 | 61.4 | 2.2 | 0 | 0 | 7.2 | 2.2 | 0 | 4.0 |
| Fracture of lower leg, including ankle | 226 | 40.3 | 40.7 | 3.1 | 2.2 | 0 | 4.4 | 2.2 | 0 | 5.8 |
| Fracture of femur | 207 | 61.4 | 33.3 | 0.5 | 0 | 0 | 0.5 | 2.4 | 0 | 1.4 |

**Includes: sexual Assault, poisoning, drowning, suffocation and Gunshot*

***These are top 10 EU diagnoses, only primary diagnosis was included, and 153 patients were missing final EU diagnosis*
